# Present Status and Perspectives on the Use of Alkylresorcinols as Biomarkers of Wholegrain Wheat and Rye Intake

**DOI:** 10.1155/2012/462967

**Published:** 2012-01-18

**Authors:** Alastair B. Ross

**Affiliations:** Department of Bioanalytical Sciences, Nestlé Research Center, Vers Chez Les Blanc, 1000 Lausanne 26, Switzerland

## Abstract

Alkylresorcinols (ARs) were first proposed as potential biomarkers of wholegrain wheat and rye intake a decade ago. Since then there has been a considerable body of research which suggests that ARs do meet most criteria of a biomarker of these foods. Results from human studies on plasma AR and their plasma and urinary metabolites strongly indicate that these compounds are responsive to whole grain wheat and rye intake and are correlated with various measures of AR consumption. This review briefly summarises work on the bioactivities of AR and focuses on aspects related to their use as biomarkers of whole grain wheat and rye intake. Evidence suggests that they thus far broadly fulfil the criteria to act as biomarkers of these cereals. However, there are still gaps in the knowledge on factors relating to the wide interindividual variation, and application to different epidemiological cohorts. Overall, ARs are highly promising biomarkers of whole grain wheat and rye intake and add to our increasing understanding of whole grains and health.

## 1. Introduction

Many epidemiological studies link a greater intake of wholegrain (WG) cereals to a decreased risk of many diet-related diseases including cardiovascular disease, obesity, type 2 diabetes, and some types of cancer [1–4]. In nutritional epidemiology, collecting valid dietary intake data, especially with food frequency questionnaires (FFQs), is challenging and remains one of the main weaknesses of this type of research [5]. For estimating WG intake, there are a number of additional challenges, including the breadth of different foods and processing methods this food category covers, and the difficulty that consumers (and researchers) have in accurately knowing the WG content of foods consumed [6]. One way of ameliorating this uncertainty around assessment of WG intake would be to use biomarkers of WG intake in conjunction with dietary assessment, and perhaps ultimately without dietary assessment (e.g., in cohorts where dietary data is not available or of questionable reliability such as blood banks and the elderly) to provide a nonsubjective estimate of intake [7]. Biomarkers of WG intake could also be of use as markers of compliance in long-term intervention studies that are being carried out in order to establish causality between increased WG intake and decreased risk of disease.

**Figure 1:**
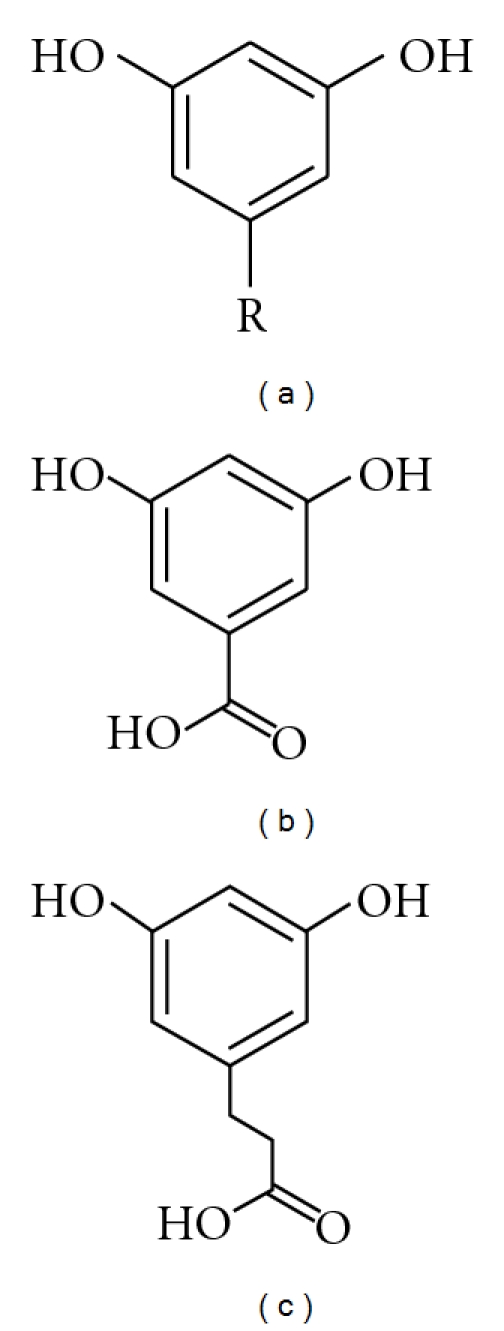
Basic structure of alkylresorcinols (a), and the two main plasma and urinary metabolites, 3,5-dihydroxybenzoic acid (b), and 3,5-dihydroxyphenylpropianoic acid (c). For the most abundant alkylresorcinols in cereals, R = C_17_H_35_–C_25_H_51_.

**Figure 2:**
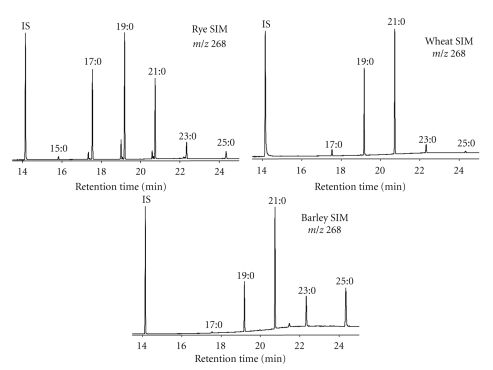
GC-MS chromatograms of the main AR containing cereals: wheat, rye, and barley. The ratio of the different odd-numbered saturated homologues varies from grain to grain but is generally conserved from variety to variety. The ratio C17 : 0/C21 : 0 can be used to determine if a cereal sample is wheat (~0.1) or rye (~1.0) and is reflected partially in plasma.

In the early part of this century, alkylresorcinols (ARs) were proposed as potential biomarkers of wholegrain wheat and rye intake [8]. ARs are 3,5-dihydroxy-phenolic lipids with an odd-numbered alkyl chain generally ranging from C15 to C25, and among food plants, only found in appreciable quantities in wheat, rye, barley, and triticale (a wheat × rye hybrid) [9] (Figure 1). The alkyl chains are mostly saturated (>80%) [9], though unsaturated, keto, and oxo-derivatives are also found, particularly in rye [10–12]. In the kernels, ARs are only found in the inner pericarp, hyaline layer, and testa [13], meaning that in food they are only present in the wholegrain or bran fraction of these cereals. The ratio of the different saturated homologues differs between the different cereals and can be used to differentiate between the three main ARs containing cereals [14] (Figure 2). The ratio of C17 : 0/C21 : 0 is approximately 0.1 in wheat (0.01 for durum wheat) and 1 in rye, and this ratio has been suggested to be a method for determining if a cereal product contains wheat, rye, or a mixture [14, 15] (Table 1). Barley has a much larger proportion of C25 : 0 compared to the other cereals, though overall has much lower total AR concentrations (40–110 *μ*g/g versus 300–1500 *μ*g/g for wheat and rye) [9, 16–18]. ARs are not found in appreciable concentrations in other food plants, though homologues C15 : 0, C17 : 1, and C17 : 2 have been found in mango flesh at low concentrations (4–17 mg/kg fresh weight) [19]. Estimates for average daily intake range from 12 mg in the United Kingdom to nearly 40 mg in Finland [20], though this may underestimate intakes at the low end of the range as the small amounts present in white wheat flour were not accounted for [16].

## 2. Bioactivity of Alkylresorcinols

Current evidence for an important bioactivity of ARs is mostly weak and based on in vitro tests, with likely activity revolving around their ability to integrate into membranes and inhibit enzymes [21]. A wide variety of bioactivities have been ascribed to AR from in vitro tests, ranging from induction of apoptosis, inhibition of lipoxygenases, and cleavage of DNA to triglyceride reduction in adipocytes [8, 12, 21, 22]. The range of effective concentrations (based on reported IC_50_) is around 3–100 *μ*mol/L. Here it is important to note that the *C*_max_ of plasma alkylresorcinols after a single meal containing 190 mg of rye AR was 3-4 *μ*mol/L [23], meaning that likely plasma concentrations are unlikely to reach a point where acute effects could be observed at “normal” intakes of 12–40 mg/d. The maximum AR concentration found in a small sample of human adipose tissues was 3.8 *μ*mol/kg [24], suggesting that AR could possibly accumulate in some tissues to relatively high concentrations and play some biological role, though significant in vitro effects on lipolysis in adipocytes were only observed at 34–38 *μ*mol/L [22].

ARs do have some antioxidant capacity, but this is weak compared to known antioxidants such as *α*-tocopherol [25, 26]. They were found to have slight antimutagenic and better antioxidant effects in membrane-based models [26]. The concentration needed to observe the inhibition of LDL oxidation was 2.5 *μ*mol/L and 75 *μ*mol/L to observe antimutagenic effects [26], whereas the average AR concentration in erythrocytes after an AR-rich diet was 315 nmol/L packed cells, and 166 nmol/L plasma in total lipoproteins [27], suggesting that strongly bioactive concentrations are unlikely in blood under normal conditions.

Only limited in vivo work has been carried out on the possible biological function of AR, with one study demonstrating that, at up to 5 g/kg feed, there is no toxic effect [28], and another demonstrated that AR could increase tissue *γ*-tocopherol concentrations via competitive inhibition of its metabolism by CYP450 enzymes [29]. Oral dosing with pure AR has not been tested in humans. While most evidence does not point to a strong bioactivity of AR, there is an increasing amount of in vitro and animal work that suggests that AR may play a role in preventing intestinal cancers. Recent studies suggest that AR are one of the main active components in the prevention of colon cancer by wheat bran and wheat bran oil in mouse and in vitro models [30–32], which is supported by previous evidence suggesting that cereal ARs have some antimutagenic and apoptotic activity [33–35], implying a mechanism for colon cancer prevention beyond fibre. This may not be the case for all types/stages of cancer, and purified AR had no effect on implanted prostate cancers in mice although rye bran did inhibit tumour growth in the same model [36]. A small case-control study with subjects with breast cancer found that plasma and urinary AR metabolites were lower in patients with breast cancer though cereal fibre intake was also lower, and it is not possible to imply causality [37]. In a larger case-control study, plasma AR did not predict lower endometrial cancer risk in Danish women [38] though, for both types of hormone-related cancers, there is no strong link between incidence and consumption of wholegrains.

## 3. Alkylresorcinols as Markers of Wholegrain Cereals in Food

ARs have been suggested to be potential markers for WG wheat and rye in food products [14] and have been used in multianalyte methods for determining the presence of difference cereal fractions in cereal foods, with AR being most indicative of the inner pericarp and testa [39, 40]. AR could also be used as a method for checking contamination of nongluten containing cereals with gluten containing cereals (wheat, rye, and barley). Even white flour contains low amounts of AR (20–50 *μ*g/g; [16]), meaning that sufficiently sensitive methods (e.g., gas chromatography-mass spectrometry (GC-MS) or high-performance liquid chromatography coupled to either CoulArray electrochemical detection (HPLC-CAED) or fluorescence detection) could be suitable for this purpose. Similarly, plasma AR could be used as a method to check for the compliance of people with coeliac disease to coeliac-free diets, as even people following WG-free diets have low plasma AR concentrations, while people avoiding all gluten containing cereals have no AR present in their plasma [41].

## 4. Alkylresorcinols as Biomarkers of Wholegrain Wheat and Rye Intake

The general criteria for an intake biomarker and how well AR meet these are outlined in Table 1. As saturated ARs are only found in the outer parts of WG wheatrye, barley, and triticale, and not in other food plants, they are good potential biomarkers of these cereals (they can also be markers for the bran intake, though generally bran alone is consumed in much lower quantities than the wholegrain and is not included in the American Association of Cereal Chemist's wholegrain definition [42]). In addition, ARs are not destroyed during food processing [9, 15] and are well absorbed in humans [43] though data from pigs and humans suggests that the percentage absorption is lower at higher doses [44, 45]. After absorption, ARs are transported in lipoproteins (mostly HDL) [27] and may be distributed and stored in some tissues, especially adipose tissue [29, 46]. ARs are metabolised via *β*-oxidation of the alkyl chain into two main metabolites: DHPPA and DHBA [47]. These metabolites can be measured in plasma and urine and are also being assessed as biomarkers of wholegrain intake [48, 49].

**Figure 3:**
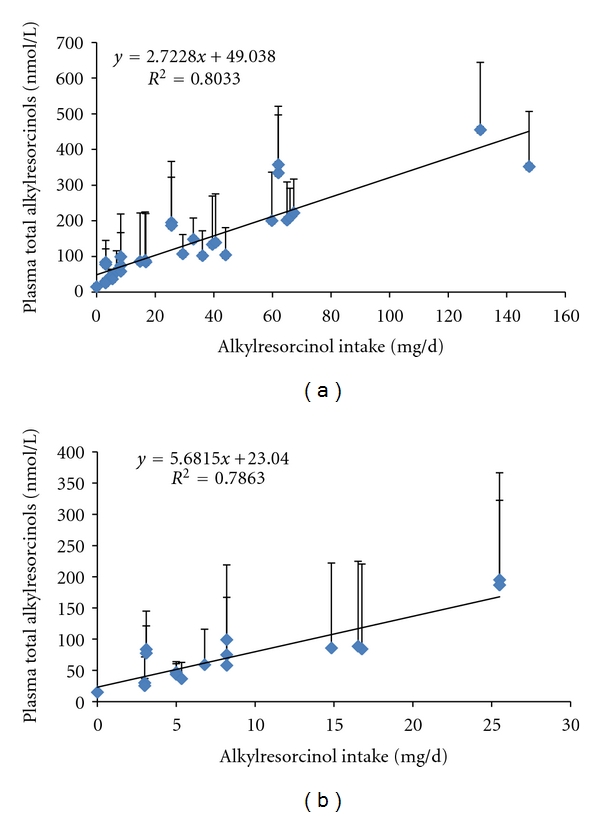
Relationship between mean AR intake and the mean of plasma AR across published studies (a). Where direct AR intake data was not provided, it was estimated from literature values if possible. Values are arithmetic means, and error bars are the standard deviation. Figure (b) uses data from studies where there has been an arm/group with an AR intake equivalent to 0–48 g of WG wheat (0–27 mg AR/d) to give an idea of the range at “normal” intakes, as well as the likely intercept for no WG intake.

**Figure 4:**
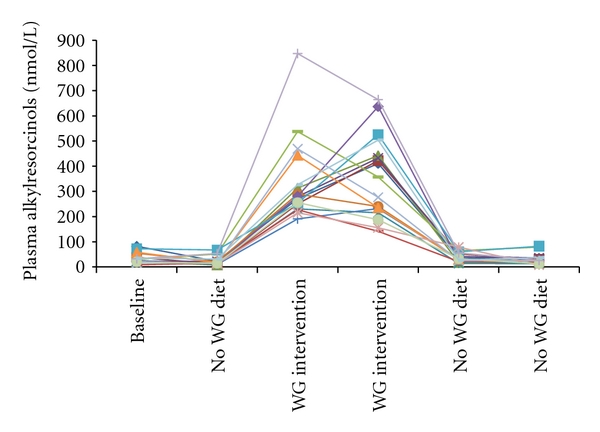
An example of interindividual variation of plasma alkylresorcinols under controlled conditions. The wholegrain intervention delivered approximately 62 mg alkylresorcinols/d. Each time point is one week apart. Data are from [51].

Kinetic considerations are important in assessing if a biomarker could be a short, medium, or long-term indicator of dietary exposure. The half-life of AR in plasma is around 5 h [23] though the exact shape of the curve may differ against a background of regular AR intake compared to a single dose after a washout [50, 51]. For the two main AR metabolites, the estimated half-life is 10–16 h in plasma [52] and 10–12 h in urine [53] though the dose used (100 mg total AR) is far greater than what would be expected for even a single day consuming WG-rich foods [20]. Landberg et al. [45] found in a dose-response study that increasing doses of AR lead to lower recoveries of AR metabolites in urine (89–45% between 22.5 and 90 mg AR/d), agreeing with previous data from pigs [44] that an increased intake does impact on absorption and metabolism, and that AR response in biological fluids may not be linear, especially at higher intakes (Figure 3). Fasting ARs do increase with regular intake, but also rapidly decrease with decreased or no intake (e.g., [27, 51, 54]; Figure 4), so that irregular intake of WG food would potentially be an important confounding factor though as cereal-based foods are generally part of the staple diet, their intake tends to be regular. One possibility for the use of AR as long-term biomarkers of WG intake is their analysis in adipose tissue [46]. While it is not known how important this pool is, nor the factors governing its turnover, it could be of use in studies where adipose tissue biopsy samples are available.

### 4.1. Methodological Considerations

ARs have been quantified in biological samples using a variety of methods. In plasma, they have been analysed using GC-MS [41, 55] and liquid chromatography-tandem mass spectrometry (LC-MS/MS) [56], erythrocytes using GC-MS [57] and GC-MS/MS [58], and in adipose tissue using GC-MS [24]. The two AR metabolites have been analysed in urine using GC-MS [47, 59] and in both plasma and urine using HPLC-CAED [48, 49]. In urine, the HPLC-CAED and GC-MS give comparable results [60]. Due to the relatively low concentrations present in plasma, MS-based methods are needed for the intact AR. GC-MS has been the main instrument used though this requires liquid-liquid extraction followed by solid phase extraction cleanup and derivitasation compared to only liquid-liquid extraction and centrifugation for normal-phase LC-MS/MS. For both methods, run times are similar (15–20 minutes injection to injection) though GC-MS has marginally greater sensitivity. MS also has the advantage that labelled internal standards can be used that are less prone to contamination or peak-overlapping issues than other types of internal standard. The use of the cheaper HPLC-CAED for analysis of metabolites for large cohorts of samples is attractive but needs to be balanced against a longer sample preparation as samples need to be deconjugated overnight, and liquid-liquid extraction is still required for plasma samples. A longer chromatographic separation and reequilibration time is needed (60 minutes per sample), greatly reducing throughput, and there is the potential for overlapping peaks; paracetamol/acetaminophen has been found to coelute with DHBA [61]. Additionally there is no information on if wheat or rye was the main source of wholegrain, if this is of interest. Ultimately antibody-based assays or similar methods will be required for AR/AR metabolite analysis to be routine in large epidemiological cohorts.

### 4.2. Studies Using AR as a Biomarker

#### 4.2.1. Intervention Studies

**Figure 5:**
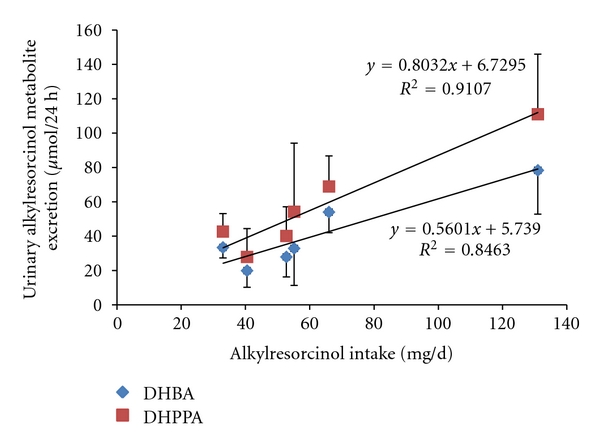
Relationship between mean AR intake and the mean of urinary AR from three published studies. Where direct AR intake data was not provided, it was estimated from literature values if possible. Values are arithmetic means, and error bars are the standard deviation.

ARs have now been measured in a number of intervention studies which now allows an overview of their performance under a variety of conditions. All studies have found that plasma AR and AR metabolites in plasma and urine are generally responsive to increased WG wheat/rye intake and that concentrations decrease rapidly on WG-free diets. The published studies are summarised in Figures 4 and 5, and Tables 2 and 3. While on average plasma ARs are highly responsive to the consumption of foods containing AR, there is a wide range of interindividual variation. Landberg et al. [54] found that repeatability, as determined using the intraclass correlation coefficient, was good under intervention conditions (ICC = 0.88 – 0.9), but less so under free-living conditions (ICC = 0.42–0.48, with one study finding a large difference between men and women) [62–64]. This variation in free-living subjects does make it difficult to classify an individual's WG wheat/rye intake with great precision based on a single sample though currently it appears as though it is a valid measure for comparing different populations, as mean plasma ARs are well correlated with mean AR intake when results from relevant studies are combined (Figure 3).

#### 4.2.2. Correlation of AR in Biological Fluids with Measurements of Wholegrain Intake

Correlations between plasma AR and various measures of their intake (AR intake, WG intake, cereal fibre intake) range between 0.25 and 0.58 (Table 2), with generally better correlations with more detailed dietary intake instruments. The studies using a general diet FFQ had the lowest correlations (<0.4), with food diaries between 0.32 and 0.52, and weighed food records and specific WG FFQ between 0.5 and 0.58. This fits with the assumption that general dietary recording methods such as FFQ are not the best instruments for collecting data on WG intake. The correlations found for plasma and urinary AR metabolites are in a similar range to the intact compounds (Table 3). While measuring urinary metabolites has an advantage over plasma samples as they are relatively unaffected by fluctuations due to different meal times (provided they are 12 or 24 h collections [65], presently there does not appear to be an advantage for either AR or their metabolites, and the metabolites are yet to be assessed in larger populations (>100 subjects) and at lower levels of AR intake (Figure 5). In the one study where the two have been compared, there were no major differences between total plasma AR or urinary AR metabolites [66]. The type of dietary exposure measurement related to AR/AR metabolite response surprisingly does not appear to be of great importance, as even very broad categories such as WG intake or rye intake lead to similar correlations to AR intake. One exception is in an intervention study based in the UK, where many subjects had low WG intake, but still varied in AR intake due to a high consumption of refined wheat foods, and correlation with AR intake was somewhat better than with WG intake (Table 2) [63].

Correlations for AR and AR metabolites are generally higher than those for *α*-carotene, *β*-cryptoxanthin, and lycopene for consumption of vegetables, fruits, and tomatoes, respectively [67, 68], and as in the case of fruits and vegetables, the choice of dietary recall method is important. Correlations could be improved by the use of absorption estimates to improve the association with estimated intake and plasma AR. Absorption estimates improved validity coefficients for serum lycopene determined using the method of triads by 50% and 100% for lycopene intake determined by 3DWFR [69]. Limited human absorption and bioavailability data exist for AR [23, 43, 45], and these studies have generally used intakes well beyond what would be expected in the general population, and more studies of this nature will be invaluable for improving the estimation of WG intake via plasma AR.

#### 4.2.3. Application of Alkylresorcinols as Surrogate Measurements of WG Intake in Epidemiological Studies

To date, only one study has used AR as a surrogate marker of WG intake. This study did not find a relationship between plasma AR and endometrial cancer incidence [38] but did find that nonfasting plasma AR and rye bread intake were moderately correlated (*r* = 0.25) [70]. This study specifically used plasma AR measurements in an attempt to improve the estimation of WG intake in this cohort. As this study used nonfasting plasma samples, there may have been greater variation, making it potentially more difficult to find associations, even when time since last meal is accounted for. The half life of AR is relatively short [23], and a single consumption event is unlikely to have a major impact on overnight fasting plasma AR concentrations but could do on a nonfasting sample. Andersson et al. found that the use of nonfasting samples leads to poor reproducibility (ICC = 0.18) [64], suggesting that they may not be ideal samples for using AR as biomarkers of WG intake. In this respect, more work is needed to compare the validity of fasting versus nonfasting plasma samples.

#### 4.2.4. Alkylresorcinols as Biomarkers of Compliance

Dietary compliance is often uncertain in dietary intervention studies; particularly, free-living studies run over long periods of time. Compliance biomarkers are sometimes used in nutrition intervention studies to support diet record collection, though often these biomarkers are directly related to health outcomes or nutrient status, for example, plasma carotenoids, lipids, and 24 h urinary nitrogen and potassium [71–73]. Compliance biomarkers not only provide an additional control of dietary compliance but can be an additional motivating factor for subjects to comply, if they know that this will be checked in their biological samples.

Plasma ARs have only been used as a compliance biomarker in a few studies to date [51, 74]. One reason for this is that there is still uncertainty around cut-off points for determining when a person has not been eating a certain quantity of WG per day, or if they are naturally low absorbers/fast metabolisers of AR. Without clear criteria for determining compliance, it is difficult to exclude subjects on the basis of AR measurements, if traditional measurements indicate that they are compliant. At the population level, it appears as though it is not difficult to distinguish between 0-1 servings of WG versus 3 servings of WG (e.g., Figure 3) using plasma AR; at an individual level, it is questionable due to the great amount of interindividual variation.  Table 4 lists the means and where possible the ranges for total plasma AR for nonwholegrain or low WG diets in the literature to date. Mean concentrations range from 33 to 84 nmol/L though, when reported, the median is often much lower than the arithmetic mean, indicating that often the mean is skewed by relatively few high concentrations, resulting in high-standard deviations. Assessing the studies with the greatest dietary control (subjects instructed to avoid other cereal foods), as well as the skewness of the data (median versus mean) and the standard deviation, it would appear that someone with a plasma AR concentration >100 nmol/L is probably eating at least some WG in their diet, and, conversely, if a subject has a plasma AR concentration of <60–70 nmol/L, then they are probably not eating any WG in the diet. By plotting the studies that have recorded low intakes of AR (<30 mg/d), a diet free of cereal AR would lead to a mean plasma AR concentration of 31 nmol/L (Figure 3(b)). There is a great need for controlled studies which will allow the determination of realistic ranges for people eating no or less than one serving of wholegrain per day, and to determine at what amount of wholegrain intake is it possible to say that they are categorically complying with the diet.

#### 4.2.5. Response of AR and AR Metabolites in Biological Fluids after Interventions

Using effect size estimates, it is possible to estimate the average response of plasma AR to a given amount of AR or WG intake. Landberg et al. [70] estimated that plasma AR would increase on average by 85 nmol/L for every 100 g of rye bread eaten in a Danish population, equating to an increase of 85 nmol/L for every 70 mg of AR consumed (based on 700 *μ*g AR/g for Danish rye bread [9, 16]). In a UK intervention study, it was estimated that 10 g of WG would lead to a 6% increase in plasma AR [63].

#### 4.2.6. Use of the Alkylresorcinol Homologue Ratios in Biological Samples

The ratio of the homologues C17 : 0 and C21 : 0 is indicative of wheat or rye in cereal samples, and this has been found to be reflected to some extent in human samples [27] although the ratio after a rye diet is a lot less than 1—usually around 0.3. This is presumed to be due to the faster metabolism of the longer chain AR [23]. In populations where both wheat and rye are eaten, it could be possible to use the C17 : 0/C21 : 0 ratio to determine if a person eats more of one of these cereals than the other. Presently there has been little specific research on the use of this ratio to determine the source of the cereal in the diet though it is clearly different between wheat-based and rye-based interventions. As the ratio in wheat is never above 0.1, it would theoretically be impossible for a subject just eating wheat to have a ratio above 0.1, so that any ratio above this in plasma would indicate at least some rye in the diet. However, this can be confounded at low concentration levels as C17 : 0, a minor homologue in wheat, may be close to the limit of quantification in plasma. In studies in populations that have rye as an important source of wholegrain, where AR concentrations have been measured (i.e., Denmark, Finland and Sweden), the ratio is generally above 0.1 [75]. However, in those populations where rye is not commonly consumed, the ratios are generally lower: 0.17 ± 0.14 for Switzerland [76], 0.06 after a 16-week WG-based intervention in the UK [63], and 0.08 ± 0.06 after a 12-week WG wheat intervention in Danish women [74] (Table 2). Together these results suggest that the C17 : 0/C21 : 0 ratio should be indicative of the source of AR beyond intervention studies and that ratios above 0.15 are probably indicative of rye intake, provided that potential analytical errors are accounted for. More understanding of the absorption and metabolism of the different homologues is required for more accurate use of the C17 : 0/C21 : 0 ratio for determining the source of AR.

#### 4.2.7. Noncereal Determinants of Plasma Alkylresorcinol Concentration

While the wide interindividual variation of plasma AR concentrations with similar intakes is well established (e.g., [51, 77]), there is still little information about what additional factors influence AR concentrations in plasma, and their metabolism. Two studies have found differences in concentrations between males and females [62, 63], but no such consistent effects have been found for age or BMI [62, 63, 70]. Under intervention conditions, while mean AR concentrations were different between the genders, the ability of AR to distinguish between different intake levels was similar [63], but ICC estimated for free-living Germans was very different, with females having much higher ICC for repeated plasma AR measurements than males [62]. Plasma lipids are correlated with plasma AR, though whether this is an independent determinant is debatable, as two studies have found that adjusting for total plasma lipids has had little or no effect on correlations with measures of AR intake [63, 70]. As ARs do not appear to play a particular role in vital bodily functions, it is unlikely that there are specific control mechanisms that would exert homeostatic control over AR concentrations in plasma or excretion in urine.

#### 4.2.8. Application of Biological Measurements of Alkylresorcinols for Dietary Recall Method Validation

While in some cases measurements of AR or AR metabolites could be used where dietary intake data does not exist or is not well suited for determining WG intake, the true potential of measuring AR in epidemiological studies lies in improving estimations of WG intake. Examples of this are the calibration of dietary recall methods, and to identify likely under- or overreporters of intake [78]. The method of triads is a widely used tool to calibrate new dietary questionnaires, using a “gold standard” method (e.g., weighed food record or 24 h recall), the questionnaire being tested, and a biomarker [79]. Because the measurement errors for the two subjective methods are correlated, the biomarker provides a crucial unbiased measurement of intake [80]. In a small study (*n* = 29) [76], the validity coefficients (a measure of “closeness” to estimated true intake) was 0.65 for plasma AR (Ross et al. unpublished observations), though larger numbers of subjects (e.g., >100) would be needed to strengthen this observation. Estimation of how well AR or AR metabolite measurements classify or rank subjects according to intake is also important to gauge their validity as biomarkers of intake [81] to know if they can reliably distinguish between extremes of WG intake.

## 5. Notes for Using Alkylresorcinols in Clinical Trials

Researchers wishing to use AR as biomarkers of WG intake, either as a surrogate marker of intake, or as a check of compliance should bear in mind the following.

- Blood is best collected on EDTA—while no studies have directly compared EDTA versus other coagulants, generally EDTA provides better stability for lipophilic compounds.
- If possible, collect cereal food samples associated with the study (if an intervention) or from the area where the study has been carried out, in order to get an estimate of AR intake. Some foods may differ from what might be expected.
- If possible, keep some check on the time since last meal. Nonfasting samples are not recommended for estimating possible WG intake, and large differences in time since last meal between subjects or time points may have an impact on the results.
- ARs are relatively responsive to changes in WG wheat/rye intake, and a one-week non-WG washout is sufficient to go from high plasma AR concentrations to low plasma AR concentrations.

## 6. Current Status and Gaps

Presently, ARs appear to be highly promising biomarkers of wholegrain wheat and rye though there are many factors that are poorly understood. Present studies find that intake of these grains still only accounts for 9–11% of the variation observed, even under relatively controlled conditions. This would suggest that unknown genetic or lifestyle factors play an even larger role in determining their concentration in individuals, even if the only dietary source is from these cereals. However, there is a strong correlation between mean concentrations and mean intake, suggesting that while ARs may be only moderate in predicting individual WG intake, they may strongly predict mean intake in larger populations.

Now that it is clear that plasma AR increases with greater WG intake, there is a need for more studies that look at the validity of AR as biomarkers at ranges of WG intake that are relevant to the intake that would be expected in general populations. More studies are also needed in non-European populations in order to assess their applicability across different types of WG intake.

## Figures and Tables

**Table 1:** Key steps for validating a biomarker, and if alkylresorcinols meet these criteria as biomarkers of alkylresorcinol containing foods (adapted from [75, 82]).

*(1) Present in wholegrain foods, but not refined foods, nor other food sources*		

Quantitative analytical methods for grains and food	GC	[9, 83]
	HPLC	[16, 84, 85]
	Colorimetry	[86–88]

Not present in other foods	In food plants, only found in wheat, rye and barley, and genetically related crops, and in low amounts in mango flesh. Very low amounts in beer and animal fat.	[9, 19, 70]

Not affected by food processing	AR stable during baking and pasta production	[9, 15]
	Limited effect of fermentation and germination in rye	[89, 90]

Variation in raw material	Wheat (350–900 *μ*g/g)	[9, 15, 91, 92]
	Rye (500–1300 *μ*g/g)	[83, 93, 94]
	Barley (30–100 *μ*g/g)	[17, 18]

*(2) Intake, absorption, distribution, metabolism, and elimination*		

Quantitative analytical methods for biological samples	GC-MS (plasma, erythrocytes, adipose tissue, urinary metabolites)	[41, 55, 57] [46, 59]
	GC-MS/MS (plasma, erythrocytes)	[58]
	LC-MS/MS (plasma)	[56]
	HPLC-CAED (metabolites)	[48, 49]

Intake	Average intake in the UK and Sweden estimated to be 12 and 23 mg/d, respectively	[20]

Absorption	Pigs: 60–79% depending on dose	[44]
	Humans: 58% ileal absorption	[43]

Distribution	Rats: negligible accumulation 100 h after a single dose	[44]
	Adipose: AR-measured in rat and human adipose	[29, 46]

Metabolism	Main AR metabolites in humans: DHBA and DHPPA	[47]
	DHBA and DHPPA also measured in human plasma	[49]
	DHPPA extensively glucuronidated in human urine	[59]

Elimination	61% and 31% of a single dose eliminated in faeces and urine in rats	[44]
	Urinary recovery 45–89% depending on dose	[45]

*(3) Dose response and pharmacokinetics*		

Dose response	Increased dose of AR leads to decreased absorption in pigs	[44]
	Urinary recovery % lower with increased AR dose	[45]

Pharmacokinetics	Pigs: *T*_max⁡_: 3 h; *T*_1/2_: 4 h	[50]
	Humans: *T*_max⁡1_: 2.8 h; *T*_max⁡2_: 6.7 h; *T*_1/2_: 4.8 h	[23]
	Plasma metabolites: *T*_max⁡_: 6 h; *T*_1/2_: 10–16 h Urinary metabolites: *T*_max⁡_: 6 h; *T*_1/2_: 10–12 h	[52, 53]

*(4) Determinants of biological concentrations, variation, and reproducibility*		

Determinants of plasma alkylresorcinol concentration	Gender: males have generally higher concentrations	[62, 63]
	Triglycerides and lipoproteins	[27, 63]
	Nonesterified fatty acids	[63]

Variation in different populations	Healthy subjects, fasting plasma	
	Mixed results for females with hormone-related cancers	[37, 70]

Reproducibility and validity	Intervention studies: good-to-moderate ICC	[54, 63]
	Free-living studies: low ICC	[62]

*(5) Application in clinical and epidemiological studies*		

Surrogate endpoint for WG intake	Endometrial cancer case-control study: no difference in nonfasting plasma AR	[38]

Validation of dietary assessment tools	WG FFQ: correlation with FFQ: 0.53	[76]

Biomarker of compliance to an intervention	WG interventions	[51, 63, 74]

**Table 2:** Correlations of plasma alkylresorcinol concentration with different measurements of wholegrain intake from previously published studies.

*N*	Gender	Country	Type of study	Dietary assessment method	Dietary exposure parameter	Correlation	*P* value	C17 : 0/C21 : 0	Reference
39	F^a^	Finland	Intervention	4DFR^b^	Rye bread intake	0.34	0.037	0.84	[95]
39	F	Finland	Intervention	4DFR	Insoluble fibre	0.39	0.013	0.84	[95]
28	F+M	Sweden	Intervention	3DFR^c^	AR	0.58	<0.001	0.30	[77]
56	F	Finland	Free-living	5DFR^d^	Cereal fibre	0.38	0.004	0.62	[66]
29	F+M	Switzerland	Free-living	3DWFR^e^	WG intake	0.57	<0.001	0.17	[76]
29	F+M	Switzerland	Free-living	WG FFQ^f^	WG intake	0.55	<0.001	0.17	[76]
360	F	Denmark	Prospective	FFQ^g^	Rye bread intake	0.25	<0.001	0.40	[70]
266	F+M	UK	Intervention	FFQ	WG intake	0.35^i^	<0.001	0.07	[63]
266	F+M	UK	Intervention	FFQ	WW^h^ intake	0.43^i^	<0.001	0.07	[63]
266	F+M	UK	Intervention	FFQ	AR intake	0.39^i^	<0.001	0.07	[63]

^a^F: female, M: male

^b^4DFR: 4-day food record

^c^3DFR: 3-day food record

^d^5DFR: 5-day food record

^e^3DWFD: 3-day weighed food diary

^f^WG-FFQ: Wholegrain food frequency questionnaire

^g^General diet food frequency questionnaire

^h^WW: Wholegrain wheat

^i^After 16-week intervention.

**Table 3:** Correlations of plasma and urinary alkylresorcinol metabolites (DHBA and DHPPA) with different measurements of wholegrain intake from previously published studies.

N	Gender	Country	Type of study	Dietary assessment method	Diet exposure parameter	AR metabolite	Plasma/urine	Correlation	*P* value	Reference
56	F^a^	Finland	Free-living	5DFR^b^	Cereal fibre	DHBA^c^	24 h urine	0.37	0.005	[66]
56	F	Finland	Free-living	5DFR	Cereal fibre	DHPPA^d^	24 h urine	0.41	0.002	[66]
56	F	Finland	Free-living	5DFR	Cereal fibre	DHBA	Plasma	0.41	<0.01	[96]
56	F	Finland	Free-living	5DFR	Cereal fibre	DHPPA	Plasma	0.46	<0.01	[96]
56	F	Finland	Free-living	5DFR	Cereal fibre	Total AR metabolites	Plasma	0.42	<0.01	[96]
60	F	Finland	Free-living	5DFR	Rye	DHBA	Plasma	0.32	<0.05	[97]
60	F	Finland	Free-living	5DFR	Rye	DHPPA	Plasma	0.39	<0.01	[97]
60	F	Finland	Free-living	5DFR	Rye	Total AR metabolites	Plasma	0.33	<0.05	[97]
60	F	Finland	Free-living	5DFR	Rye	DHBA	24 h urine	0.52	<0.001	[97]
60	F	Finland	Free-living	5DFR	Rye	DHPPA	24 h urine	0.44	<0.001	[97]
60	F	Finland	Free-living	5DFR	Rye	Total AR metabolites	24 h urine	0.48	<0.001	[97]

^a^F: female

^b^5DFR: 5-day food record

^c^DHBA: 3,5-dihydroxybenzoic acid

^d^DHPPA: 3,5-dihydroxyphenylpropionoic acid.

**Table 4:** Plasma AR concentrations when subjects have consumed low or essentially AR-free diets.

*N*	Gender	Country	AR intake (mg/d)	Intervention type	Duration of intervention period (weeks)	Median	Mean	SD	Range	Reference
39	F^a^	FI	5.34^b^	Replace all bread with intervention breads	8		36.6	26.2	10.9–55.8^c^	[95]
15	F+M	FI	3^b^	Replace all bread with intervention breads	1		25–30	12–41	5.5–171	[27]
28	F+M	SE	6.8	All cereal foods provided	6		59	57	9–220	[77]
17	M	SE	8.2	Replace all cereal foods	6	33	72	101	17–410	[54]
17	F+M	CH	5	Fully controlled diet	2	40	44	17	27–89	[51]
34	F	DK	3.1^d^	Replace part of cereals in diet	12	61	78	43.7	16–246	[74]
266	F+M	UK	17	WG consumption < 30 g/d	0	69.5	84.3	136	10–875	[63]
16	F+M	SE	WG-free diet	Avoid all WG foods	1		60–68	33–37	23–178	[45]
17	F+M	CH	WG-free diet	Avoid all WG foods	1	19–32	25–38	13–21	7–82	[51]

^a^ F: female, M: male

^b^Estimated intake from refined wheat bread intake

^c^Excludes outliers

^d^Amount provided by intervention, not total diet.
